# Single Cell Raman Spectroscopy Deuterium Isotope Probing for Rapid Antimicrobial Susceptibility Test of *Elizabethkingia* spp.

**DOI:** 10.3389/fmicb.2022.876925

**Published:** 2022-05-03

**Authors:** Shuying Yuan, Yanwen Chen, Kaicheng Lin, Lin Zou, Xinrong Lu, Na He, Ruijie Liu, Shaoxing Zhang, Danfeng Shen, Zhenju Song, Chaoyang Tong, Yizhi Song, Wenhong Zhang, Li Chen, Guiqin Sun

**Affiliations:** ^1^School of Medical Technology and Information Engineering, Zhejiang Chinese Medical University, Hangzhou, China; ^2^CAS Key Laboratory of Bio-Medical Diagnostics, Suzhou Institute of Biomedical Engineering and Technology, Chinese Academy of Sciences, Suzhou, China; ^3^Department of Medical Microbiology and Parasitology, Key Laboratory of Medical Molecular Virology of Ministries of Education and Health, School of Basic Medical Sciences, Fudan University, Shanghai, China; ^4^Department of Emergency Medicine, Zhongshan Hospital, Fudan University, Shanghai, China; ^5^Department of Infectious Diseases, Huashan Hospital, Shanghai Medical College, Fudan University, Shanghai, China

**Keywords:** *Elizabethkingia* spp., single cell Raman spectroscopy deuterium isotope probing, carbon-deuterium ratio, antimicrobial susceptibility test, minimum inhibitory concentration

## Abstract

Nosocomial infection by multi-drug resistance *Elizabethkingia* spp. is an emerging concern with severe clinical consequences, particularly in immunocompromised individuals and infants. Efficient control of this infection requires quick and reliable methods to determine the appropriate drugs for treatment. In this study, a total of 31 *Elizabethkingia* spp., including two standard strains (ATCC 13253 and FMS-007) and 29 clinical isolates obtained from hospitals in China were subjected to single cell Raman spectroscopy analysis coupled with deuterium probing (single cell Raman-DIP). The results demonstrated that single cell Raman-DIP could determine antimicrobial susceptibility of *Elizabethkingia* spp. in 4 h, only one third of the time required by standard broth microdilution method. The method could be integrated into current clinical protocol for sepsis and halve the report time. The study also confirmed that minocycline and levofloxacin are the first-line antimicrobials for *Elizabethkingia* spp. infection.

## Introduction

The emergence and widespread distribution of antimicrobial-resistant bacteria have led to increasing concerns regarding potential environmental and public health risks. The crisis of antimicrobial-resistant bacteria has been attributed to the overuse and misuse of antimicrobials (Ventola, 2015). Therefore, it is important for clinicians to know the drug resistance patterns of pathogens and use suitable antimicrobials. In clinical practice, detecting bacterial drug resistance relies on phenotypic antimicrobial susceptibility testing (AST) approaches, such as “minimum inhibitory concentration” (MIC) determined by the broth microdilution method (BMD; Balouiri et al., 2016; Brauner et al., 2016). At least 16–18 h are needed to detect antimicrobial effects on bacterial population growth in isolated colonies by this method. Rapid detection of microbial antimicrobial susceptibility could ensure the selection of effective antimicrobials and reduce the total antimicrobial consumption (Kerremans et al., 2008).

Raman spectroscopy (RS) is a label-free, fast, and non-destructive biochemical phenotype technology that is used to detect the vibrational modes of molecules (Tao et al., 2017). Single cell RS provides a biochemical “fingerprint” of individual cells, which reflects cell physiological and metabolic states (Song et al., 2017). It has been applied to identify bacterial strains (Kloss et al., 2013; Schroder et al., 2013; Tang et al., 2013; Colnita et al., 2017) and to detect physiological changes during the treatment with antimicrobials (Wichmann et al., 2019; Hilton et al., 2020). It was reported that *Listeria monocytogenes* with different susceptibilities to sakacin P had different Raman spectra in 2006 (Oust et al., 2006). Single cell RS analysis of different pathogens, including methicillin-resistant *Staphylococcus aureus* (MRSA), *Enterococcus faecium*, *Enterococcus faecalis*, *Escherichia coli*, *Klebsiella pneumoniae*, *Pseudomonas aeruginosa*, *Acinetobacter baumannii*, *Serratia marcescens*, and *Lactococcus lactis*, has been carried out (Liu et al., 2009; Assmann et al., 2015; Wang et al., 2016a; Dekter et al., 2017; Kwiatkowski et al., 2019; Li et al., 2019; Fu et al., 2020).

It has been discovered that metabolically active microorganisms can incorporate deuterium (D) into the cells *via* the NADH/NADPH electron transport chain, producing a newly formed carbon-deuterium (C-D) band in single cell RS analysis coupled with deuterium probing (single cell Raman-DIP; Berry et al., 2015; Wang et al., 2016b; Song et al., 2017; Tao et al., 2017). The occurrence of a C-D band around 2,040–2,300 cm^–1^ has been recognized as an antimicrobial resistance biomarker when bacteria were exposed to antimicrobials and D_2_O (Song et al., 2017). Recently, single cell Raman-DIP was proposed to achieve fast AST for pathogens such as *E. coli*, *K. pneumoniae*, *Streptococcus mutans*, *Lactobacillus fermentum*, *E. faecalis*, and *S. aureus* (Tao et al., 2017; Hong et al., 2018; Yang et al., 2019; Zhang et al., 2020; Yi et al., 2021). However, in previous studies, pathogens were treated with antimicrobials at up to 8 or 9 different concentrations. A full panel of AST would require laborious work to collect Raman spectra for single cells of hundreds of treatments.

Hospital infection associated with *Elizabethkingia* spp. is an emerging clinical concern characterized by multi-drug resistance and severe clinical consequences. Many cases of *Elizabethkingia* spp. infections have been reported as part of outbreaks in the state of Wisconsin (United States), London (United Kingdom), and Mauritius (Issack and Neetoo, 2011; Moore et al., 2016; Perrin et al., 2017). The infection of *Elizabethkingia* spp. has been reported and received great interesting (Frank et al., 2013; Jean et al., 2014; Sun et al., 2015; Chen et al., 2020). *Elizabethkingia* spp. are a group of gram-negative, non-ferment pathogens that are responsible for a panel of diseases, including meningitis, sepsis, bacteremia, pneumonia, and neutropenic fever (da Silva and Pereira, 2013; Lau et al., 2016; Lin et al., 2016; Burnard et al., 2020). So far, the potential of single cell Raman-DIP application for fast AST for *Elizabethkingia* spp. has not been demonstrated.

In this study, the optimized single cell Raman-DIP was applied to two standard strains of *Elizabethkingia* spp. (ATCC 13253 and FMS-007) and 29 clinical isolates. The AST readout of *Elizabethkingia* spp. was determined by single cell Raman-DIP within 4 h and highly consistent results were shown with the gold standard. The optimized approach only required 3 or 4 concentrations based on the breakpoints of guidelines M100 (CLSI, 2019) of treatments for each antimicrobial, and thus promoted the efficiency of the AST and achieved the goal of clinical application. Single cell Raman-DIP could also be directly applied to positive blood culture samples with as low as 10^6^ CFU/mL of *Elizabethkingia* spp. The possibility of applying single cell Raman-DIP for the clinical diagnosis of *Elizabethkingia* spp. was demonstrated.

## Materials and Methods

### Microorganisms and Growth Conditions

Thirty clinical *Elizabethkingia* spp. strains collected in China and American Type Culture Collection (ATCC 13253) were used in this study (Sun et al., 2014, 2015). The identification and antimicrobial susceptibility of 31 strains were tested using the automated VITEK 2 Compact system (BioMérieux, France) with the gram-negative identification card (GN) and AST-GN16 card, respectively. All strains were grown aerobically in trypticase soy broth (TSB; Sigma-Aldrich, United States) at 37^°^C overnight and then seeded on a blood agar plate (BioMérieux, France) for single colonies.

### Minimum Inhibitory Concentration of *Elizabethkingia* spp. Determined by Broth Microdilution Method

A standard assay (CLSI, 2019) was performed in a 96-well microplate (Bio-Kont, China). The tested bacterial colonies on each blood agar plate were aseptically transferred into Mueller-Hinton (MH) broth (BD Biosciences, United States), and a homogenous suspension with a density equivalent to 0.5 McFarland’s standard was prepared. The bacterial suspension was then diluted with MH broth at a ratio of 1:200. The bacterial solution (100 μL) was inoculated into wells with sequentially diluted antimicrobials and incubated at 37^°^C at 180 rpm for 16 h. Aztreonam, cefepime, imipenem, ticarcillin/clavulanic acid, amikacin, tobramycin, minocycline, and levofloxacin were used in this study. The concentrations of the eight antimicrobials were based on the breakpoints of other non-Enterobacteriaceae listed in the guidelines M100 (CLSI, 2019). MIC values and the results of antimicrobial susceptibility were interpreted based on the guidelines M100 (CLSI, 2019).

### Antimicrobial Susceptibility Detected by Optimized Single Cell Raman-DIP

D_2_O (99% D atom, Sigma-Aldrich, United States) labeling was performed as previously reported (Berry et al., 2015; Song et al., 2017). In this study, the three breakpoint-concentrations of each antimicrobial were tested, instead of multi-proportion dilution method. The benefit was that the number of tests was reduced, which increased detection efficiency and reduced time. Three to five isolated colonies selected from each blood agar plate were subjected to single cell Raman-DIP. Bacterial suspension was prepared for AST. One hundred microliters of inoculated bacterial solution was added to each 96-well plate with standard concentrations of antimicrobial suggested in the guidelines M100 (CLSI, 2019) and incubated at 37°C at 180 rpm for 1 h. Sixty-six microliters D_2_O was added to each well, and then incubated for two more hours. The cells were washed with sterile deionized water three times and resuspended in 20 μL of sterile deionized water. The cell suspension (2.5 μL) was transferred onto an aluminum-coated slide which was placed on the *x-y-z* motorized stage of a confocal Raman spectrometer (P300, Hooke Instruments, China) with 100 × magnifying dry objective (Olympus, Japan). The laser power used on the samples was approximately 5 mW. At least 20–30 single cell Raman spectra were recorded by a –70°C cooled charge-coupled device (PIXIS 100 B, Princeton instruments, United States) with an integration time of 8 s per spectrum.

### Single Cell Raman-DIP for Bloodstream Infection Samples

The FMS-007 strain was used as an example to simulate bloodstream infection samples. The bacterial contents in the simulated samples ranged from 10^6^ to 10^7^. Each simulated sample (1 mL) was collected and diluted ten times (1 mL sample was added to 9 mL sterile normal saline). The diluted samples (10 mL) were injected into a serum separator tube with gel and clot activator (Becton, Dickinson and Company, United States), statically incubated at 37°C for 1 h, and then centrifuged to remove the majority of blood cells at 2,500 rpm for 2 min. Bacteria were present in the supernatant, washed three times with sterile water, and resuspended in 1 mL of sterile water. AST was performed as described previously. This protocol was reviewed and approved by the Medical Ethics Committee of Zhejiang Chinese Medical University (Approval No. 20211202-4).

### Data Analysis

The range of the Raman spectra was extracted from 400 to 3,400 cm^–1^. The cosmic rays and baselines were corrected in Labspec6 (Horiba JY, Tokyo, Japan) with 10-degree linear baseline fitting algorithm. Then, the spectra were normalized using a total area below all the peaks within the spectral range. The Raman spectra for carbon-deuterium (C-D) peaks (2,040–2,300 cm^–1^) and carbon-hydrogen (C-H) peaks (2,800–3,100 cm^–1^) were obtained. The C-D ratio was calculated (C-D/C-D+C-H) and normalized (C-D ratio of treated group minus C-D ratio of no deuterium and no antimicrobials controls). The impact of treatment was determined by the relative metabolic rate (defined as the ratio of normalized C-D of the treatment to that of the no drug control; Yi et al., 2021). Graphpad Prism was used for statistical analysis. Statistical tests performed were indicated in figure legend. Numerical data were shown as mean ± SEM. *P* values of less than 0.001 were considered statistically significant.

## Results

### Antimicrobial Susceptibility Testing of 31 *Elizabethkingia* spp. Strains by Automated Drug Sensitivity Analysis System

The AST results of 31 strains were listed in Table 1 and they were consistent with previous reports (Burnard et al., 2020; Wang et al., 2020). Collectively, our previous studies of around 150 clinical isolates indicated that the resistance rates of aztreonam, ampicillin, cefazolin, ceftriaxone, imipenem, amoxicillin/clavulanic acid, tobramycin, and nitrofurantoin were >97% in the tested strains (Sun et al., 2014; Li et al., 2020). However, ciprofloxacin and levofloxacin all demonstrated susceptibility rates of >60%.

**TABLE 1:** The antimicrobial susceptibility of 31 *Elizabethkingia* spp. strains.

Antimicrobial	S (%)	I (%)	R (%)
ATM	0 (0.00)	0 (0.00)	31 (100.00)
AMP	0 (0.00)	0 (0.00)	31 (100.00)
CFZ	0 (0.00)	0 (0.00)	31 (100.00)
FOX	5 (16.13)	0 (0.00)	26 (83.87)
CRO	0 (0.00)	0 (0.00)	31 (100.00)
IPM	0 (0.00)	0 (0.00)	31 (100.00)
AMC	0 (0.00)	0 (0.00)	31 (100.00)
AMK	0 (0.00)	0 (0.00)	31 (100.00)
GEN	0 (0.00)	3 (9.68)	28 (90.32)
TOB	0 (0.00)	0 (0.00)	31 (100.00)
TIM	6 (19.35)	22 (70.97)	3 (9.68)
CIP	24 (77.42)	2 (6.45)	5 (16.13)
LVX	26 (83.87)	1 (3.23)	4 (12.90)
NIT	0 (0.00)	0 (0.00)	31 (100.00)

*S, susceptible; I, intermediate; R, resistant; ATM, aztreonam; AMP, ampicillin; CFZ, cefazolin; FOX, cefoxitin; CRO, ceftriaxone; IPM, imipenem; AMC, amoxicillin/clavulanic acid; AMK, amikacin; GEN, gentamicin; TOB, tobramycin; TIM, tigecycline; CIP, ciprofloxacin; LVX, levofloxacin; and NIT, nitrofurantoin.*

### Rapid Antimicrobial Susceptibility Testing of Imipenem for *Elizabethkingia* spp. by Single-Cell Metabolic Activity

**FIGURE 1:**
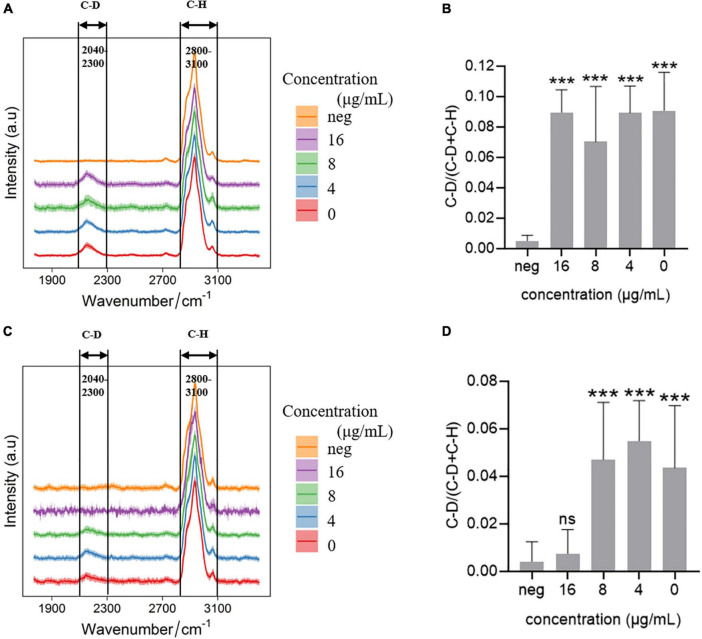
Raman spectra of cells treated with imipenem **(A)** C-D and C-H band in FMS-007; **(B)** the C-D ratio of spectra in **(A)**; **(C)** C-D and C-H band in TZ-3; **(D)** the C-D ratio of spectra in **(C)**; and neg: without D_2_O and imipenem. Data were shown as mean ± SEM of neg and the other four group. ***P < 0.001; ns, no significance.

Single cell Raman-DIP was used to measure the metabolic activity of bacteria at the single-cell level (Berry et al., 2015). *Elizabethkingia* spp. strains were incubated in MH medium containing 40% heavy water for 2 h, the deuterium in the medium was incorporated into the cellular biomass and produced a new peak at 2,040–2,300 cm^–1^ (C-D band) on a background of 2,800–3,100 cm^–1^ (C-H band; Figures 1A,D). Imipenem was used as an example to detect metabolism under antimicrobial treatment using single cell Raman-DIP. After exposure to imipenem, the intensity of the C-D band in strain FMS-007 remained at the same level as the drug-free control, in which no imipenem was added (Figure 1A), and the C-D ratio (Figure 1B) were calculated. The number of biological replicates in each group was shown in supplementary data (Supplementary Table 2). This indicated that bacteria were still metabolically active under imipenem up to 16 μg/mL, resulting in an MIC of >16 μg/mL. Since 16 μg/mL was the resistant breakpoint of *Elizabethkingia* spp. for imipenem, strain FMS-007 was regarded as resistant to imipenem. The C-D band of strain TZ-3 was not detected when imipenem was present at a concentration of 16 μg/mL (Figures 1C,D), indicating that the bacterial metabolic activity was inhibited and the MIC was determined to be 16 μg/mL. The results showed that single cell Raman-DIP could be used to detect microbial metabiotic activity and antimicrobial susceptibility of *Elizabethkingia* spp.

### The Antimicrobial Susceptibility Testing of *Elizabethkingia* spp. by Single Cell Raman-DIP and the Cutoff Value of Relative Metabolic Rate

**FIGURE 2:**
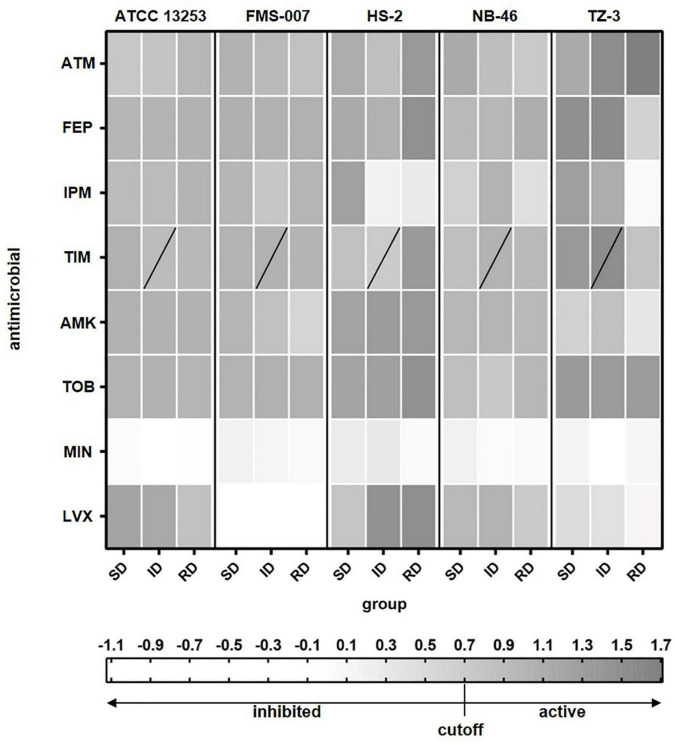
The relative metabolic rate of the five *Elizabethkingia* spp. strains determined by Raman-DIP. The antimicrobial concentrations of group SD, ID, and RD were obtained from the M100 guidelines (CLSI, 2019). SD, susceptible breakpoint corresponds to antimicrobial concentration; ID, intermediate breakpoint corresponds to antimicrobial concentration; RD, resistant breakpoint corresponds to antimicrobial concentration; ATM, aztreonam; FEP, cefepime; IPM, imipenem; TIM, ticarcillin/clavulanic acid; AMK, amikacin; TOB, tobramycin; MIN, minocycline; and LVX, levofloxacin. TIM was composed of two intermediate breakpoints (the data of lower concentration of intermediate breakpoints was shown).

The antimicrobial susceptibility of five *Elizabethkingia* spp. strains to eight antimicrobials was tested using single cell Raman-DIP and classical BMD methods. The results of single cell Raman-DIP and BMD were summarized in Figure 2 and Table 2, respectively. The breakpoints of antimicrobial susceptibility according to the guidelines M100 (CLSI, 2019) were used in this study.

**TABLE 2:** The AST of five *Elizabethkingia* spp. strains.

	ATCC13253		FMS-007		HS-2		NB-46		TZ-3		
Antimicrobial	BMD	Ram	BMD	Ram	BMD	Ram	BMD	Ram	BMD	Ram	BMD/Ram
ATM	R	R	R	R	R	R	R	R	R	R	R/R
FEP	I	R	S	R	I	R	I	R	S	R	S/R, I/R
IPM	R	R	R	R	I	I	R	R	R	R	R/R, I/I
TIM	R	R	R	R	R	R	R	R	R	R	R/R
AMK	R	R	R	R	R	R	I	R	R	R	R/R
TOB	R	R	R	R	R	R	R	R	R	R	R/R
MIN	S	S	S	S	S	S	S	S	S	S	S/S
LVX	R	R	S	S	R	R	I	R	S	S	S/S, R/R

*AST, antimicrobial susceptibility testing; BMD, broth microdilution method; Ram, single cell Raman-DIP; S, susceptible; I, intermediate; R, resistant; ATM, aztreonam; FEP, cefepime; IPM, imipenem; TIM, ticarcillin/clavulanic acid; AMK, amikacin; TOB, tobramycin; MIN, minocycline; and LVX, levofloxacin.*

To compare the results from Raman-DIP and BMD, we used 3 cutoff values for relative metabolic rate, i.e., 0.6, 0.7, and 0.8. Antibiotic treated single cells with relative metabolic rate below the cutoff value were considered to be inhibited by the antibiotic. The cutoff value of 0.7 was shown to give the highest consistency with conventional BMD approach (Supplementary Table 1), and thus the corresponding concentration of relative metabolic rate < 0.7 was used to interpret the results of antimicrobial susceptibility. The results of seven antimicrobials were consistently detected by single cell Raman-DIP and classical BMD methods, including aztreonam, imipenem, ticarcillin/clavulanic acid, amikacin, tobramycin, minocycline, and levofloxacin. However, the AST results of the two methods were different for cefepime. Five strains were all resistant to cefepime tested by single cell Raman-DIP, but the results of testing by BMD were sensitive or intermediate.

In the AST results tested by single cell Raman-DIP, all five strains were resistant to five antimicrobials (a total of eight antimicrobials tested), including aztreonam, cefepime, ticarcillin/clavulanic acid, amikacin, and tobramycin (Figure 2). ATCC 13253, HS-2, NB-46, and TZ-3 were sensitive to minocycline, while FMS-007 was intermediate. Except for strain HS-2, which was intermediate, they were resistant to imipenem. FMS-007 and TZ-3 were sensitive to levofloxacin, but ATCC 13253, HS-2, and TZ-3 were resistant. This implied that minocycline and levofloxacin were probably most suitable drugs for *Elizabethkingia* spp. infection.

### The Antimicrobial Susceptibility Testing of Minocycline and Levofloxacin of *Elizabethkingia* spp. by Single Cell Raman-DIP

**FIGURE 3:**
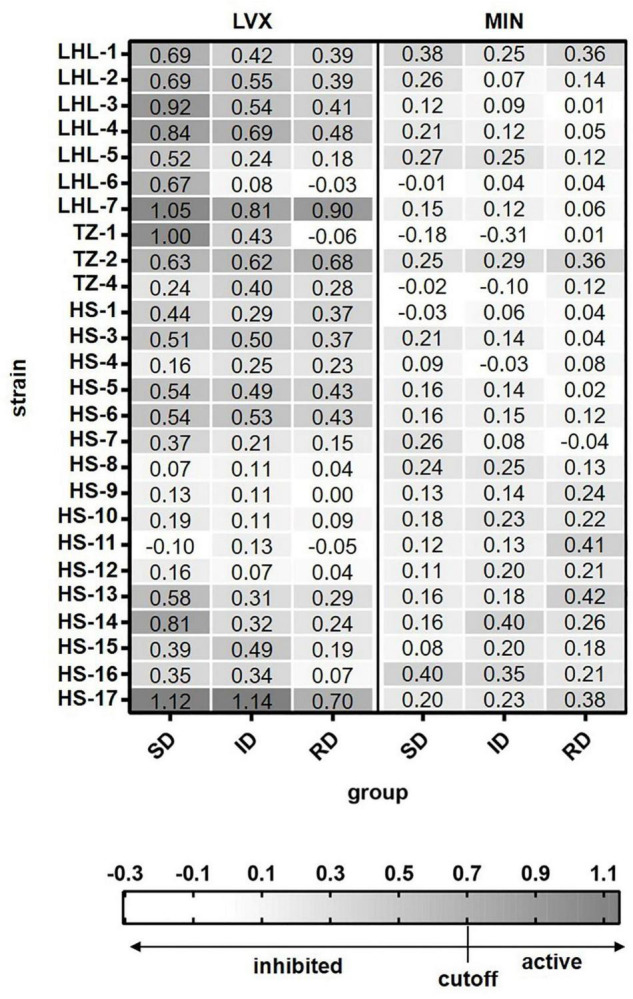
The relative metabolic rate of 26 *Elizabethkingia* spp. strains determined by Raman-DIP. The antimicrobial concentrations of group SD, ID, and RD were obtained from the M100 guidelines (CLSI, 2019). SD, susceptible breakpoint corresponds to antimicrobial concentration; ID, intermediate breakpoint corresponds to antimicrobial concentration; RD, resistant breakpoint corresponds to antimicrobial concentration; MIN, minocycline; and LVX, levofloxacin. The relative metabolic rates of each concentration were shown in each grid.

Based on the above results, single cell Raman-DIP could be used to detect metabiotic activity of *Elizabethkingia* spp. Because *Elizabethkingia* spp. were more sensitive to minocycline and levofloxacin, the two antimicrobials were tested against the other 26 *Elizabethkingia* spp. The cutoff value of 0.7 was used to interpreted the AST of other 26 *Elizabethkingia* spp. The results from single cell Raman-DIP and classic BMD methods were shown in Figure 3 and Table 3. The AST of minocycline tested by BMD (Supplementary Table 3) and single cell Raman-DIP was 100% consistent, while the consistency of levofloxacin was approximately 87.10%.

**TABLE 3:** The AST of LVX and MIN of *Elizabethkingia* spp. strains.

Strain	LVX		MIN	
	AST by Ram	AST by BMD	AST by Ram	AST by BMD
LHL-1	S	S	S	S
LHL-2	S	S	S	S
LHL-3	I	S	S	S
LHL-4	I	S	S	S
LHL-5	S	S	S	S
LHL-6	S	S	S	S
LHL-7	R	R	S	S
TZ-1	I	S	S	S
TZ-2	S	S	S	S
TZ-4	S	S	S	S
HS-1	S	S	S	S
HS-3	S	S	S	S
HS-4	S	S	S	S
HS-5	S	S	S	S
HS-6	S	S	S	S
HS-7	S	S	S	S
HS-8	S	S	S	S
HS-9	S	S	S	S
HS-10	S	S	S	S
HS-11	S	S	S	S
HS-12	S	S	S	S
HS-13	S	S	S	S
HS-14	I	S	S	S
HS-15	S	S	S	S
HS-16	S	S	S	S
HS-17	R	R	S	S

*AST by BMD, the results of antimicrobial susceptibility tested by broth microdilution method; AST by Ram, the results of antimicrobial susceptibility tested by single cell Raman-DIP; MIN, minocycline; and LVX, levofloxacin.*

### The Antimicrobial Susceptibility Testing of Bloodstream Infection Samples by Single Cell Raman-DIP

**FIGURE 4:**
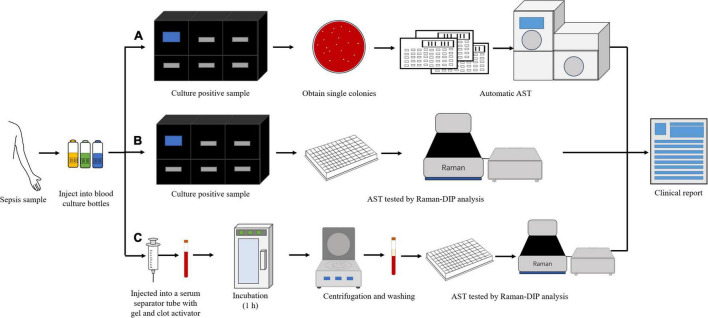
The different procedures of AST for clinical blood samples. **(A)** the procedure of current clinical routine method; **(B)** the procedure of current clinical routine method combined with single cell Raman-DIP; and **(C)** the procedure of this study.

**FIGURE 5:**
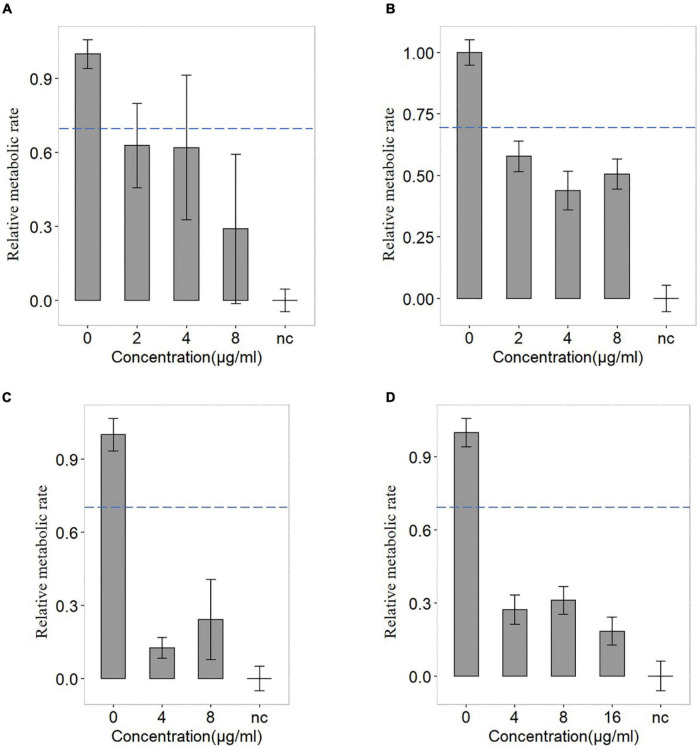
The antimicrobial susceptibility of levofloxacin and minocycline of FMS-007. **(A)** the blood sample containing 10^6^ CFU/mL treated with levofloxacin; **(B)** the blood sample containing 10^7^ CFU/mL treated with levofloxacin; **(C)** the blood sample containing 10^6^ CFU/mL treated with minocycline; and **(D)** the blood sample containing 10^7^ CFU/mL treated with minocycline. The dotted lines indicated the cutoff value at 0.7 of the relative metabolic rates.

Bloodstream infection caused by *Elizabethkingia* spp. has been reported (Reed et al., 2020; Kuo et al., 2021). In this study, the blood samples were diluted ten times and injected into a serum separator tube with gel and clot activator to remove blood cells (Figure 4). It was a much more convenient and effective way to remove blood cells than using ammonium-chloride-potassium (ACK) buffer and repeated washing (Han et al., 2020; Yi et al., 2021). The relative metabolic rate directly measured from bacterial single-cells in the blood sample were shown in Figure 5. The number of biological replicates in each group of simulated blood sample was shown in supplementary data (Supplementary Tables 4, 5). The results indicated that the pathogens in the blood samples were susceptible to minocycline and levofloxacin since their relative metabolic rate were below 0.7 when the concentrations were at the breakpoints concentration for susceptible. These results were the same as the clinical isolates results (Figure 2 and Table 2). The metabolic activity of bacteria in the blood sample with bacterial concentration as low as 10^6^ CFU/mL (except for one case where single cells were not spotted in bacterial contents of 10^6^ treated with 16 μg/mL minocycline) can be determined by Raman-DIP without strain sub-culture. These results demonstrated that this method could be directly integrated into current clinical protocol for sepsis and halve the report time for AST (Figure 4).

## Discussion

Establishing a quick and reliable drug resistance assay is an urgent clinical need for medical practice and public health. In this study, single cell Raman-DIP analysis was applied to measure the fingerprint and metabolic response of *Elizabethkingia* spp. to a panel of antimicrobials. The isolates of *Elizabethkingia* spp. responded with high sensitivity to minocycline and levofloxacin, as confirmed by the 4-h single cell Raman-DIP procedure. This was consistent with previous reports (Sun et al., 2014; Burnard et al., 2020; Li et al., 2020; Wang et al., 2020; Lin et al., 2021). Our study on *Elizabethkingia* spp. was an addition to the previous single cell Raman-DIP application studies.

In a few cases, inconsistent susceptibility results between single cell Raman-DIP and BMD methods were obtained. In addition to cefepime, the other seven antimicrobials showed an overall consistency rate of 94% for single cell Raman-DIP. Notably, in all inconsistent cases, the results given by single cell Raman-DIP were resistant while those obtained by the BMD method were sensitive or intermediate, which means they were no major errors. These results met the FDA requirements (category agreement ≥ 90%, minor error ≤ 10.0%, major error ≤ 3.0%, and very major error ≤ 1.5%; Zhang et al., 2020). Inconsistent results were shown for cefepime between single cell Raman-DIP and BMD. Cefepime works by inhibiting penicillin-binding proteins essential for cell wall formation (Yahav et al., 2020). A possible explanation for cefepime was that the replication of bacteria under the treatment of cefepime at concentrations higher than the BMD was inhibited, but the cells were still metabolically active. Recently, it has been revealed that growth-arrested bacteria may still exhibit metabolic activity in *Mycobacterium tuberculosis* (Ehrt et al., 2018) and persistent bacteria treated with antimicrobials (Orman and Brynildsen, 2013). Single cell metabolic activity assessment *via* single cell Raman-DIP could provide new insights into antimicrobial administration to patients.

In this study, it was demonstrated that the results of antimicrobial susceptibility were similar between single cell Raman-DIP and BMD methods. It took 4 h to obtain the AST results by single cell Raman-DIP, which was 4–5 times faster than the BMD method. The results demonstrated that AST determined by single cell Raman-DIP was comparable to BMD, and single cell Raman-DIP was a practical complementation for BMD.

The AST results of patients with severe infection could be tested by using a combination of with classical BMD method and single cell Raman-DIP (Figure 4). If the bacterial contents of patient’s blood samples were more than 10^6^ CFU/mL, the AST results could be obtained within 6 h by single cell Raman-DIP, which was providing a rapid basis for clinical treatment. It was demonstrated that single cell Raman-DIP combined with removing cells was a direct method for performing the AST of blood samples, including for cases with bacteremia caused by *Elizabethkingia* spp. and other bacteria. This method could be extended to samples of cerebrospinal fluid and body fluids.

Infection caused by *Elizabethkingia* spp. is a rare but life-threatening medical condition (Frank et al., 2013). A critical challenge for clinicians is to prescribe a useful antimicrobial, although strains are multi-drug resistant. In this study, we demonstrated that single cell Raman-DIP could achieve a rapid and reliable AST of *Elizabethkingia* spp. in 4 h. Thus, great potential in clinical studies and diagnosis of *Elizabethkingia* spp. were demonstrated by single cell Raman-DIP techniques.

Quick and efficient clinical management of rare infection is an unmatched clinical need for individual and global heath and deserves more research efforts. Previous studies concluded that *Elizabethkingia* spp. was an emerging rare infection, which was susceptible mainly to levofloxacin and minocycline treatment. In this translational oriented study, we applied single cell Raman-DIP to 31 *Elizabethkingia* spp. strains, and the results generated in 6 h were consistent with the conclusion in early reports. The methods could also be directly applied to positive blood culture samples. In addition, our study also indicated that this method could also be extended to pathogens isolated from blood, cerebrospinal fluid, and other body fluids, including *Elizabethkingia* spp. The establishment of this quick method can help clinicians to make quick decisions on which one is more efficient for a specific case.

## Data Availability Statement

The original contributions presented in the study are included in the article/Supplementary Material, further inquiries can be directed to the corresponding authors.

## Ethics Statement

The studies involving human participants were reviewed and approved by the Medical Ethics Committee of Zhejiang Chinese Medical University. The patients/participants provided their written informed consent to participate in this study.

## Author Contributions

SY, YC, and KL drafted and wrote the manuscript. GS, LC, WZ, and YS critically reviewed the manuscript and improved it. LZ, XL, NH, RL, SZ, DS, ZS, and CT participated in manuscript correction. All authors gave final approval and agreed to be accountable for all aspects of the work.

## Conflict of Interest

The authors declare that the research was conducted in the absence of any commercial or financial relationships that could be construed as a potential conflict of interest.

## Publisher’s Note

All claims expressed in this article are solely those of the authors and do not necessarily represent those of their affiliated organizations, or those of the publisher, the editors and the reviewers. Any product that may be evaluated in this article, or claim that may be made by its manufacturer, is not guaranteed or endorsed by the publisher.
