# Supplementary material for: Single Cell Raman Spectroscopy Deuterium Isotope Probing for Rapid Antimicrobial Susceptibility Test of Elizabethkingia spp
Source: Front Microbiol. 2022 May 3;13:876925. doi: 10.3389/fmicb.2022.876925 (PMC9113537; doi:10.3389/fmicb.2022.876925)
Supplement: Supplementary file 1 [file Table_1.docx]

Table S1 The AST of five *Elizabethkingia* spp. strains with different cutoff value

| Antimicrobial | ATCC13253 | | |  | FMS-007 | | |  | HS-2 | | |  | NB-46 | | |  | TZ-3 | | |
| --- | --- | --- | --- | --- | --- | --- | --- | --- | --- | --- | --- | --- | --- | --- | --- | --- | --- | --- | --- |
|  | Cutoff  =0.6 | Cutoff  =0.7 | Cutoff  =0.8 |  | Cutoff  =0.6 | Cutoff  =0.7 | Cutoff  =0.8 |  | Cutoff  =0.6 | Cutoff  =0.7 | Cutoff  =0.8 |  | Cutoff  =0.6 | Cutoff  =0.7 | Cutoff  =0.8 |  | Cutoff  =0.6 | Cutoff  =0.7 | Cutoff  =0.8 |
| ATM | R | R | R |  | R | R | R |  | R | R | R |  | R | R | R |  | R | R | R |
| FEP | R | R | R |  | R | R | R |  | R | R | R |  | R | R | R |  | R | R | R |
| IPM | R | R | R |  | R | R | R |  | I | I | I |  | R | R | R |  | R | R | R |
| TIM | R | R | R |  | R | R | R |  | R | R | R |  | R | R | R |  | R | R | R |
| AMK | R | R | R |  | R | R | R |  | R | R | R |  | R | R | R |  | R | R | R |
| TOB | R | R | R |  | R | R | R |  | R | R | R |  | R | R | R |  | R | R | R |
| MIN | S | S | S |  | S | S | S |  | S | S | S |  | S | S | S |  | S | S | S |
| LVX | R | R | R |  | S | S | S |  | R | R | R |  | R | R | R |  | S | S | S |

S, susceptible; I, intermediate; R, resistant; ATM, aztreonam; FEP, cefepime; IPM, imipenem; TIM, ticarcillin/clavulanic acid; AMK, amikacin; TOB, tobramycin; MIN, minocycline; LVX, levofloxacin

Table S2 The number of biological replicates in each group of Figure 1

| Strain | The number of biological replicates | | | | |
| --- | --- | --- | --- | --- | --- |
|  | Neg | 16 μg/mL | 8 μg/mL | 4 μg/mL | 0 μg/mL |
| FMS-007 | 32 | 31 | 34 | 35 | 34 |
| TZ-3 | 35 | 5 | 35 | 31 | 33 |

Table S3 The MIC of levofloxacin and minocycline by BMD

| Strain | MIC of levofloxacin (μg/mL) | MIC of minocycline (μg/mL) |
| --- | --- | --- |
| LHL-1 | S(1) | S(0.5) |
| LHL-2 | S(1) | S(1) |
| LHL-3 | S(1) | S(1) |
| LHL-4 | S(1) | S(0.5) |
| LHL-5 | S(1) | S(0.5) |
| LHL-6 | S(1) | S(0.5) |
| LHL-7 | R(8) | S(0.5) |
| TZ-1 | S(1) | S(0.5) |
| TZ-2 | S(0.5) | S(1) |
| TZ-3 | S(0.25) | S(0.5) |
| TZ-4 | S(0.5) | S(0.5) |
| HS-1 | S(1) | S(0.5) |
| HS-2 | R(16) | S(0.5) |
| HS-3 | S(1) | S(1) |
| HS-4 | S(1) | S(1) |
| HS-5 | S(0.5) | S(1) |
| HS-6 | S(1) | S(1) |
| HS-7 | S(1) | S(0.25) |
| HS-8 | S(0.5) | S(1) |
| HS-9 | S(1) | S(1) |
| HS-10 | S(1) | S(1) |
| HS-11 | S(1) | S(1) |
| HS-12 | S(0.5) | S(0.5) |
| HS-13 | S(1) | S(1) |
| HS-14 | S(1) | S(1) |
| HS-15 | S(1) | S(1) |
| HS-16 | S(1) | S(1) |
| HS-17 | R(16) | S(0.5) |
| FMS-007 | S(0.25) | S(0.25) |
| NB-46 | I(4) | S(1) |
| ATCC13253 | R(8) | S(1) |

S, susceptible; I, intermediate; R, resistant

Table S4 The number of biological replicates in each group of simulated blood sample (levofloxacin)

| Bacterial concentration (CFU/mL) | The number of biological replicates | | | | |
| --- | --- | --- | --- | --- | --- |
|  | Neg | 8 μg/mL | 4 μg/mL | 2μg/mL | 0 μg/mL |
| 10^6^ | 27 | 13 | 12 | 13 | 25 |
| 10^7^ | 23 | 27 | 27 | 27 | 30 |

Table S5 The number of biological replicates in each group of simulated blood sample (minocycline)

| Bacterial concentration (CFU/mL) | The number of biological replicates | | | | |
| --- | --- | --- | --- | --- | --- |
|  | Neg | 16 μg/mL | 8 μg/mL | 4μg/mL | 0 μg/mL |
| 10^6^ | 27 | / | 8 | 7 | 25 |
| 10^7^ | 23 | 27 | 29 | 35 | 30 |
